# Serum and salivary ferritin and Hepcidin levels in patients with chronic periodontitis and type 2 diabetes mellitus

**DOI:** 10.1186/s12903-018-0524-4

**Published:** 2018-04-10

**Authors:** Lin-Na Guo, Yan-Zong Yang, Yun-Zhi Feng

**Affiliations:** Department of Stomatology, The Second Xiangya Hospital, Central South University, 139 Renmin Middle Road, Changsha, 410011 Hunan China

**Keywords:** Hepcidin, Ferritin, Chronic periodontitis, Type 2 diabetes mellitus, Saliva, Serum

## Abstract

**Background:**

Iron disorder and abnormal expression of hepcidin play important roles in many diseases, but it is still unclear in chronic periodontitis (CP) and type 2 diabetes mellitus (T2DM). We aimed to assess ferritin and hepcidin levels in serum and saliva of CP patients with or without T2DM.

**Methods:**

Serum and unstimulated whole saliva samples were collected from 88 participants, who were categorized into 4 groups based on the presence or absence of CP or T2DM. Demographics and general health parameters were recorded. Full-mouth clinical periodontal parameters including probing pocket depth, clinical attachment loss, bleeding index, and plaque index were recorded. Chemiluminescence microparticle immunoassay and enzyme-linked immunosorbent assay were used to detect ferritin and hepcidin concentrations, respectively, in serum and saliva.

**Results:**

Serum ferritin and hepcidin levels in the CP and CP with T2DM groups were higher than in the control group (*P* < 0.05). Serum hepcidin and serum ferritin are linear correlated (*P* < 0.001). Serum hepcidin/ferritin values in the CP with T2DM group were significantly lower than those in the T2DM and control groups. Moreover, salivary ferritin levels in the CP and T2DM groups were higher than those in the control group (*P* < 0.05). There was positively correlation between salivary ferritin and serum ferritin (*P* = 0.017). Hepcidin concentrations were relatively low in saliva.

**Conclusions:**

These results suggest that iron overload and hepcidin inadequacy existed in CP with T2DM patients. Salivary ferritin might provide a reference for body iron load.

**Trial registration:**

ChiCTR-ROC-17012780

## Background

Chronic periodontitis (CP) is a bacteria-induced chronic inflammatory disease of tooth-supporting tissues [1]. Type 2 diabetes mellitus (T2DM) is characterized by chronic hyperglycemia caused by impaired insulin secretion and/or insulin resistance [2]. An association between CP and T2DM has been established, which is bidirectional [3]. Periodontitis has been accepted as a T2DM risk factor (odds ratio [OR] =1.5–2.1), which is mainly due to the oral chronic inflammatory condition leading to T2DM development by altering insulin resistance [4, 5]. It is known that T2DM affect the occurrence and development of periodontal disease by reducing resistance to infection [6, 7]. Although impaired immune response, microangiopathy, varying oral microflora, and disorders in collagen metabolism have proved to play key roles in the association between DM and periodontitis [8–10], the understanding is still incomplete and warrants further exploration. Studies have shown that majority of key periodontopathogens possess haemolytic activity, which lead to iron excess by the dissolution of erythrocyte [11]. Iron excess by such periodontal pathogens may lead to increased local iron concentrations and eventually cause iron disorder.

Iron is an indispensable nutrient for the human body; it is essential to maintain systemic iron homeostasis because iron disorder in the body can lead to various clinical diseases [12]. Recent evidence suggests that increased body iron stores play a role in the pathogenesis of T2DM. Yeap et al. detected serum ferritin, iron, and transferrin saturation among 1834 men and 2351 women and reported that higher serum ferritin levels were independently associated with DM. Since ferritin is commonly used as a marker of body iron stores, this study suggested that increased body iron stores is a risk factor for DM [13]. Excessive systemic iron can cause oxidative stress injury in hepatocytes and pancreatic β cells, forming the basis of diabetes, which may lead to insulin resistance, reduction in insulin secretion, and eventually, development of T2DM [14, 15]. In addition, it has been reported that insulin can directly up-regulate hepcidin expression in hepatocellular carcinoma (HepG2) cells by enhancing signal transducers and activators of transcription 3 (STAT3) protein synthesis and DNA binding activity [16], indicating that insulin disorder in T2DM can lead to inadequate hepcidin concentrations. Evidence suggests to us that insulin resistance accompanied by inadequate hepcidin levels can progress to overt diabetes through more dysfunction of cells via iron overload [17]. Hepcidin, a key hormone synthesized in the liver, can regulate iron homeostasis [18]; it can prevent iron efflux from enterocytes, macrophages, and hepatocytes into the plasma by inducing internalization and degradation of the iron exporter ferroportin in these cells [19], which indicates that the iron overload is further aggravated by inadequate hepcidin in T2DM. Hepcidin expression can be regulated by iron-mediated pathways through signaling of JAK/STAT and BMP/SMAD [20]. In CP patients, iron disorder might also exist. However, levels of ferritin and hepcidin are still lack of investigation in CP patients with or without T2DM. Thus, we detected concentrations of ferritin and hepcidin and the hepcidin/ferritin ratio in serum and saliva of CP patients with or without T2DM. This may be useful for further studies to explore the association between DM and periodontitis concerning iron disorder.

Iron stores can be detected using several techniques including liver biopsy [21], iron stains on bone marrow trephine biopsy [22], and serological detection. Serum ferritin level is now widely used in clinical practice and is considered the most convenient and cost-effective method to detect iron load [13]. And also, Serum hepcidin has been proved to be an indicator of iron load [23]. Moreover, the serum hepcidin/ferritin ratio has been used to evaluate iron metabolism of the body as it represents both body iron load and adequacy of hepcidin production for a given iron load [24]. Saliva, as a potential fluid for monitoring health and disease, has received increasing attention; its collection is noninvasive, and its utilization has high repeatability and is safe and inexpensive [25]. Although saliva and serum may contain similar components such as ferritin and hepcidin [26, 27], levels of constituents in saliva can be affected by the synthesis and secretion of parotid acinar cells [28], and might thus inconsistent with serum. To our knowledge, no study has been conducted to determine whether saliva could provide reference for serum in detecting ferritin and hepcidin levels and hepcidin/ferritin ratios in CP patients with or without T2DM.

## Methods

### Selection and characteristics of study population

After receiving approval from the Ethical Committee of the Second Xiangya Hospital of Central South University, we recruited 22 T2DM patients, 22 CP patients, 22 CP patients with T2DM, and 22 healthy individuals; the age of the participants ranged 40–83 years. All participants provided a written informed consent, and all steps of the clinical examination and sampling procedures were explained to each participant. Participants were diagnosed with T2DM by specialist physicians at the hospital according to the American Diabetes Association criteria [29]. A single examiner performed periodontal examination of all participants by using a manual periodontal probe (UNC15; Hu-Friedy, Chicago, IL, USA). According to American Academy of Periodontology, CP was diagnosed if ≥30% periodontal bone loss with teeth having a clinical attachment level (CAL) of ≥5 mm and a periodontal probing depth (PD) of ≥5 mm at one or more sites on the teeth at multiple sites of all four quadrants of the mouth [30]. Full-mouth periapical radiographs were taken to determine the level of periodontal bone loss in the patients. For detailed evaluation of the periodontal status, bleeding index (BI) and plaque index (PI) were also recorded [31, 32]. The criteria for enrolment included (i) patients with T2DM who were diagnosed with T2DM for > 1 year; (ii) no history of receiving professional periodontal treatment during the past 6 months; (iii) no use of antibiotics or steroidal and nonsteroidal anti-inflammatory medications during the past 3 months; and (iv) no treatment with immunosuppressive chemotherapy, no current acute illness, and no ongoing pregnancy or lactation.

Data regarding demographic and clinical characteristics, including age, body mass index (BMI), fasting blood glucose (FBG), triglyceride (TG), cholesterol (CHOL), high-density lipoprotein (HDL), low-density lipoprotein (LDL), alanine transaminase (ALT), and aspartate transaminase (AST), were recorded in physical examination reports of all participants. Clinical registration information of this study could be found on the International Clinical Trials Registry Platform (http://apps.who.int/trialsearch/Trial2.aspx?TrialID=ChiCTR-ROC-17012780).

### Collection and storage of biological samples

Before periodontal examination, unstimulated whole saliva samples were collected using a standard spit method according to the description by Navazesh [33]. Participants were requested to refrain from eating, drinking, and performing basic oral hygiene for 2 h before sample collection. Saliva sample was transferred from the plastic container to a centrifuge tube and then centrifuged at 4 °C at 13000 rpm for 20 min. Saliva samples contain sputum or blood would be discarded. Blood samples were collected from the antecubital fossa through venipuncture by using a 20-gauge needle with a 5-mL syringe. All collections were performed by well-trained nurses at the nursing station. Serum was separated from blood by centrifuging at 3000 rpm for 5 min. Blood samples which occurred hemolysis would be gave up. Each saliva or serum sample was given a tracking number and stored at − 80 °C until further analysis.

### Laboratory assays

Ferritin and hepcidin were tested in saliva and serum samples on the same day by using chemiluminescence microparticle immunoassay (CMIA) and enzyme-linked immunosorbent assay (ELISA), respectively. Ferritin assay was performed according to the manufacturer’s instructions described in the assay procedure. A two-step immunoassay was required. Ferritin present in the sample binds to antiferritin-coated microparticles. After washing, antiferritin acridinium conjungate was added. Subsequently, Pre-Trigger and Trigger Solutions were added to the reaction mixture; the resulting chemiluminescent reaction was measured as relative light units (RLUs). A direct relationship exists between ferritin concentrations in the sample and RLUs detected by the ARCHITECT i optical system. Hepcidin concentrations in saliva and serum were determined using an ELISA kit (CUSABIO Inc., Wu Han, China) according to the manufacturer’s protocol. Ferritin and hepcidin levels in serum and saliva were presented as ng/mL.

### Statistical analysis

Data analysis was performed using the SPSS statistical program (Version 17.0; SPSS, Chicago, IL, USA). We compared characteristics of participants in each group, including age, FBG, BMI, TG, CHOL, LDL, and AST by using analysis of variance (ANOVA), while HDL, ALT were compared using the nonparametric Kruskal–Wallis test. Constituent ratio of gender was analyzed by Chi-square test. As a result of the skewed distributions of periodontal parameters, non-parametric tests kruskal-Wallis were used, when we analyzed periodontal condition. The constituent ratio of gender was analyzed using chi-square test. Test of normality showed that log serum ferritin, serum hepcidin, 1/(serum hepcidin/ferritin), log salivary ferritin, 1/salivary hepcidin, and log (salivary hepcidin/ferritin) were in accordance with the normal distribution. Thus, analysis of covariance (ANCOVA) was used both for comparing levels of ferritin, hepcidin, and hepcidin/ferritin in serum and saliva among each group and for controlling the effect of age. We analyzed the relationship between serum hepcidin and serum ferritin; serum ferritin and salivary ferritin by curve fitting. In addition, stepwise linear regression analysis was used to explore risk factors of serum ferritin, hepcidin, and hepcidin/ferritin that could be entered into the model. *P* values < 0.05 represented statistical significance.

## Results

### Characteristics and clinical periodontal parameters in subjects

Table 1 summarizes the demographic characteristics of study participants: the mean participant age in the control, CP, T2DM, and CP with T2DM groups was 52.45 ± 10.01, 58.09 ± 9.97, 56.45 ± 11.80, and 62.82 ± 10.72 years, respectively. Fasting blood glucose levels were significantly higher in patients with T2DM than in those without T2DM (*P* < 0.05). However, no significant intergroup differences were noted in terms of sex, BMI, TG, CHOL, HDL, LDL, ALT, and AST (*P* > 0.05).

**Table 1:** Characteristics and periodontal parameters of participants

	Control	CP	T2DM	CP + T2DM	*P*-value
Age (in years, Mean ± SD)	52.45 ± 10.01	58.09 ± 9.97	56.45 ± 11.80	62.82 ± 10.72	0.017^a^*
Gender, % (*n*)					
Males	64% (14)	73% (16)	68% (15)	77% (17)	0.779^b^
Females	36% (8)	27% (6)	32% (7)	23% (5)	
FBG (mg/dL, Mean ± SD)	5.26 ± 0.55	5.27 ± 0.56	8.09 ± 2.22	7.64 ± 2.32	< 0.001^a^*
BMI (Mean ± SD)	24.43 ± 2.33	25.85 ± 3.51	25.48 ± 3.45	25.00 ± 2.34	0.471^a^
TG (mmol/L, Mean ± SD)	1.95 ± 0.89	2.82 ± 4.66	2.27 ± 1.59	1.90 ± 1.14	0.435^a^
CHOL (mmol/L, Mean ± SD)	5.00 ± 1.08	4.62 ± 0.85	4.44 ± 1.02	4.47 ± 1.18	0.284^a^
HDL (mmol/L, Median (IQR))	1.17 (1.03, 1.51)	1.10 (0.93, 1.18)	1.10 (0.97, 1.35)	1.17 (0.94, 1.23)	0.511^c^
LDL (mmol/L, Mean ± SD)	2.88 ± 0.72	2.61 ± 0.82	2.52 ± 0.78	2.59 ± 1.03	0.523^a^
ALT (u/L, Median(IQR))	20.00 (14.03, 29.28)	20.50 (13.10, 26.10)	20.80 (15.33, 29.65)	20.55 (15.55, 25.30)	0.992^c^
AST (u/L, Mean ± SD)	21.87 ± 4.95	22.29 ± 7.62	21.00 ± 6.43	22.40 ± 6.33	0.887^a^
PD (Mean ± SD)	2.00 ± 0.71	2.80 ± 0.45	2.00 ± 0.65	3.02 ± 0.27	< 0.001^c^*
CAL (Mean ± SD)	0.08 ± 0.20	3.04 ± 0.32	0.26 ± 0.34	4.07 ± 0.17	< 0.001^c^*
BI (Mean ± SD)	0.18 ± 0.54	1.55 ± 0.64	0.37 ± 0.59	1.82 ± 0.47	< 0.001^c^*
PI (Mean ± SD)	1.00 ± 0.48	2.23 ± 0.55	1.28 ± 0.57	2.61 ± 0.29	< 0.001^c^*

*T2DM* type 2 diabetes mellitus, *CP* chronic periodontitis, *CP + T2DM* chronic periodontitis with type 2 diabetes mellitus, *SD* standard deviation, *IQR* interquartile range, *FBG* fasting blood glucose, *BMI* body mass index, *TG* triglyceride, *CHOL* cholesterol, *HDL* high density lipoprotein, *LDL* low density lipoprotein, *ALT* alanine transaminase, *AST* aspartate transaminase, *PD* probing pocket depth, *CAL* clinical attachment loss, *PI* plaque index, *BI* bleeding index

*P*_1_: Control vs CP; *P*_2_: Control vs T2DM + CP; *P*_3_: CP vs T2DM; *P*_4_: T2DM vs T2DM + CP

^a^Analysis of variance (ANOVA)

^b^Chi-square test

^c^Kruskal-Wallis test

^*^Significantly different *P* < 0.05

Periodontal parameters including PD, CAL, BI, and PI of each group are enlisted in Table 1. On comparing these parameters between both groups at a time, values of PD, CAL, BI, and PI were higher in CP patients (*P* < 0.05).

### Measurement of ferritin, hepcidin, and hepcidin/ferritin levels in serum and saliva

**Fig. 1:**
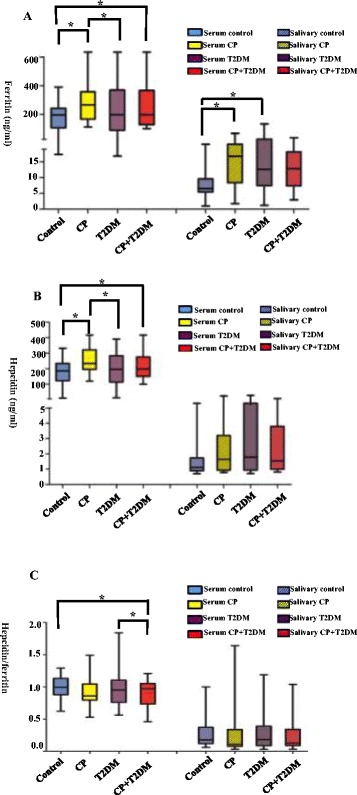
Box diagrams showing concentrations of ferritin, hepcidin, and hepcidin/ferritin (ng/mL, median [IQR]) in serum and saliva among the control, CP, T2DM, and T2DM with CP groups. Blue boxes represent control group participants, yellow boxes represent CP participants, purple boxes represent T2DM participants, and red boxes represent CP with T2DM participants. Median and interquartile range was presented by boxes, and maximum and minimum values were indicated using bars of each group. One indicator of both serum and saliva was put into one image. Shaded boxes represent indicators of saliva detection, whereas no shadows represent those of serum detection. Serum ferritin and hepcidin levels were significantly higher in the CP and CP with T2DM groups than in the control group (**a**, **b**, P* < 0.05). In addition, serum ferritin and hepcidin concentrations were significantly higher in the CP group than in the T2DM group (**a**, **b**, P* < 0.05). The change trend of serum hepcidin was similar to that of serum ferritin. The serum hepcidin/ferritin value was significantly lower in the CP with T2DM group than in the T2DM and control groups (**c**, P* < 0.05). Detection of saliva samples revealed higher ferritin levels in the CP and T2DM groups (**a**, *P* < 0.05). The salivary hepcidin levels were relatively low (**b**). Significant differences are indicated by asterisk (*, *P* < 0.05)

**Fig. 2:**
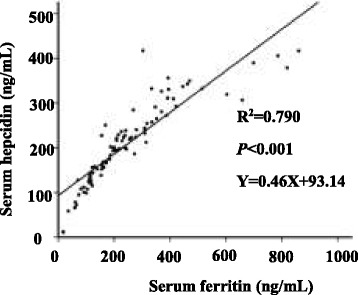
linear correlations between serum hepcidin levels and serum ferritin concentrations. We used the serum ferritin level as X, and the serum hepcidin level as Y. Dots of the graph represent the detected concentration value. Lines of the graph represent the linear correlation between them (R^2^ = 0.790, *P* < 0.001). The equation is presented in Fig. 2 (Y = 0.46X + 93.14).

Concentrations of ferritin, hepcidin, and hepcidin/ferritin were represented as median and interquartile range (Table 2, Fig. 1). As some of the raw data were skewed, log and reciprocal transformations were performed, which are also presented as mean and standard deviation in Table 2. Serum ferritin levels in the CP (*P*_1_ = 0.008) and CP with T2DM (*P*_2_ = 0.015) groups were higher than those in the control group. The CP group exhibited higher serum ferritin compared with the T2DM group (*P*_3_ = 0.036). The change trend of serum hepcidin was similar to that of serum ferritin. Serum hepcidin levels were higher in the CP (*P*_1_ = 0.003) and CP with T2DM (*P*_2_ = 0.036) groups than in the control group. Moreover, serum hepcidin levels were higher in the CP group than in the T2DM group (*P*_3_ = 0.04). The serum hepcidin/ferritin value was significantly lower in the CP with T2DM group (mean = 0.88) than in the T2DM and control groups (mean = 1.00 for both). Serum hepcidin levels were positively correlated with levels of serum ferritin (R^2^ = 0.790, *P* < 0.001, Fig. 2). On the other hand, salivary ferritin levels were significantly higher in the CP (median = 16.74 ng/mL) and T2DM (median = 12.61 ng/mL) groups than in the control group (median = 6.50 ng/mL). The hepcidin concentrations in saliva were relatively low. There was no correlation between salivary ferritin level and salivary hepcidin level (*P* > 0.05, data not shown).

**Table 2:** Levels of ferritin, hepcidin and hepcidin/ferritin in serum and saliva

	Control	CP	T2DM	CP + T2DM	*P*-value
Serum ferritin (ng/ml, Median (IQR))	196.2 (106.6, 241.6)	265.1 (166.1, 358.5)	196.3 (88.8, 369.6)	197.7 (128.9, 366.6)	*P*_1_ = 0.008^a^* P_2_ = 0.015^a^* *P*_3_ = 0.036^a^*
log serum ferritin (Mean ± SD)	2.20 ± 0.32	2.42 ± 0.20	2.23 ± 0.40	2.38 ± 0.31	
Serum hepcidin (ng/ml, Mean ± SD)	179.37 ± 79.86	249.56 ± 77.15	197.92 ± 103.04	218.36 ± 94.05	*P*_1_ = 0.003^a^* P_2_ = 0.036^a^* *P*_3_ = 0.040^a^*
Serum hepcidin/ferritin (Mean ± SD)	1.00 ± 0.18	0.95 ± 0.26	1.00 ± 0.31	0.88 ± 0.24	*P*_2_ = 0.007^a^* *P*_4_ = 0.015^a^*
Salivary ferritin (ng/ml, Median (IQR))	6.50 (5.49, 9.56)	16.74 (8.44, 23.93)	12.61 (7.47, 26.16)	12.81 (7.42, 18.13)	*P*_1_ = 0.008^a^* P_5_ = 0.014^a^*
log salivary ferritin (Mean ± SD)	0.83 ± 0.32	1.14 ± 0.36	1.11 ± 0.46	1.06 ± 0.30	
Salivary hepcidin (ng/ml, Median (IQR))	1.12 (0.89, 1.73)	1.64 (0.93, 3.19)	1.79 (0.93, 5.14)	1.54 (0.99, 3.80)	*P* = 0.307^a^
1/(salivary hepcidin) (Mean ± SD)	0.84 ± 0.38	0.66 ± 0.44	0.62 ± 0.44	0.63 ± 0.36	
Salivary hepcidin/ferritin (Median (IQR))	0.17 (0.12, 0.37)	0.11 (0.08, 0.34)	0.18 (0.90, 0.39)	0.13 (0.92, 0.34)	*P* = 0.939^a^
log (salivary hepcidin/ferritin) (Mean ± SD)	−0.69 ± 0.34	− 0.77 ± 0.51	−0.72 ± 0.42	− 0.77 ± 0.38	

*P*_1_: Control vs CP; *P*_2:_ Control vs T2DM + CP; *P*_3_: CP vs T2DM; *P*_4_: T2DM vs T2DM + CP; *P*_5:_ Control vs T2DM

^a^Analysis of covariance (ANCOVA)

^*^Significantly different *P* < 0.05

### Absence or presence of CP can influence serum ferritin and hepcidin levels and hepcidin/ferritin value

We selected serum concentrations of ferritin and hepcidin and value of hepcidin/ferritin as dependent variables and considered gender, age, BMI, FBG, TG, CHOL, HDL, LDL, ALT, AST, absence or presence of CP, absence or presence of T2DM, and duration of T2DM as independent variables (X_1_–X_13_, Table 3). Results of multiple stepwise linear regression on serum ferritin revealed that gender, age, and absence or presence of CP could be entered into the model (Y = 503.687 + 110.537X_11_–4.991X_2_–100.828X_1_). Regarding serum hepcidin, the results showed that gender, age, HDL, and absence or presence of CP could be entered into the model (Y = 359.086 + 47.604X_11_–30.911X_7_–1.946X_2_–54.76X_1_). Regarding the serum hepcidin/ferritin value, results revealed that age and absence or presence of CP could be entered into the model (Y = 0.717–0.143X_11_ + 0.008X_2_).

**Table 3:** Multiple linear regression analysis of serum ferritin, hepcidin, and hepcidin/ferritin

	Model	b	S.E.	b’	t	*P*
Serum ferritin	Constant	503.687	108.718		4.633	< 0.001*
	Gender	−100.828	37.012	−0.265	− 2.724	0.008*
	Age	−4.991	1.582	−0.315	−3.154	0.002*
	absence or presence of CP	110.537	35.156	0.316	3.144	0.002*
Serum hepcidin	Constant	359.086	58.876		6.099	< 0.001*
	Gender	−54.760	19.875	−0.275	−2.755	0.007*
	Age	−1.946	0.819	−0.235	−2.376	0.020*
	HDL	−30.911	15.367	−0.203	−2.012	0.048*
	absence or presence of CP	47.604	18.327	0.260	2.598	0.011*
Serum hepcidin/ferritin	Constant	0.717	0.141		5.097	< 0.001*
	Age	0.008	0.002	0.354	3.391	0.001*
	absence or presence of CP	−0.143	0.052	−0.287	−2.752	0.007*

X_1_: gender (1 = male, 2 = female); X_2_: age (year); X_3_: BMI; X_4_: FBG (mg/dl); X_5_: TG (mmol/l); X_6_: CHOL (mmol/l); X_7_: HDL (mmol/l); X_8_: LDL (mmol/l); X_9_:ALT (u/l); X_10_:AST (u/l); X_11_: absence or presence of CP (1 = non-CP; 2 = CP); X_12_: absence or presence of T2DM (1 = non-DM; 2 = DM); X_13_: duration of T2DM (0 = non-T2DM, 1 = 1–5 years, 2 = 6–10 years, 3 = more than 10 years)

Stepwise method used; ^*^Significantly different *P* < 0.05

### Curve correlations between serum ferritin concentration and salivary ferritin level

**Fig. 3:**
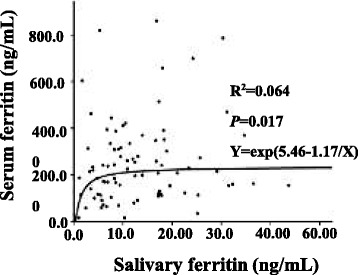
Curve correlations between serum ferritin concentrations and salivary ferritin levels. We used salivary ferritin levels as X, and serum ferritin levels as Y. Dots of the graph represent the detected concentration of ferritin. Lines of the graph represent the fitted curves. The correlation was observed between them (R^2^ = 0.064, *P* = 0.017). The equation is presented in Fig. 3 (Y = exp. (5.46–1.17/X))

We performed curve fitting between serum ferritin concentration and salivary ferritin level. The results showed that there was correlation between them (R^2^ = 0.064, *P* = 0.017, Fig. 3), furthermore, the level of serum ferritin increased with salivary ferritin (Y = exp. (5.46–1.17/X)).

## Discussion

To our knowledge, no previous study has explored concentrations of ferritin and hepcidin and the hepcidin/ferritin ratio in serum and saliva of CP patients with or without T2DM. The key findings of the present study were that patients with CP and T2DM exhibited significantly higher serum ferritin and hepcidin levels and lower serum hepcidin/ferritin ratio compared with controls. Absence or presence of CP was a significant predictor of serum ferritin concentration and the hepcidin/ferritin ratio. In addition, there was correlation between serum ferritin concentration and salivary ferritin level. Thus, these results suggested that iron overload and hepcidin inadequacy existed in CP with T2DM patients. CP might be a potential risk factor for iron overload and hepcidin inadequacy. Besides, salivary ferritin might provide a reference for body iron load.

Iron is required in the body for several biological processes, but it is also harmful when in excess [34]. Iron overload can be detected by ferritin which plays a crucial role in iron storage and recycling [13]. A previous study reported increased serum ferritin levels in patients with CP [35], which is consistent with our results. Moreover, it has been reported that Increased serum ferritin levels might be associated with the severity of CP [36]. While S. Latha et al. found no difference in levels of serum ferritin between the CP group and the control group [37], which may be due to the differences in race, sample size, and inclusion standards. Moreover, in the present study, patients with CP and T2DM exhibited higher serum ferritin levels compared with the controls. These findings suggest existed iron overload in CP and CP with T2DM patients. Given that iron overload inhibits bone formation, Mandalunis et al. evaluated the effects of iron on alveolar bone of rats and found that iron overload led to decreased interradicular bone volume [38]; this indicated that iron accumulation leads to loss of alveolar bone and thus aggravates periodontitis. Iron overload also has been confirmed as an independent factor that leads to the development of T2DM by causing oxidative stress injury in hepatocytes and pancreatic β cells [16]. Therefore, measures to regulate iron overload are crucial in CP patients with or without T2DM. Moreover, in the present study, patients with CP exhibited higher serum ferritin levels than did those with T2DM; this can be explained by the existence of periodontal pathogens, predominantly gram-negative anaerobes, which has been confirmed leading to increased ferritin level after their infection [39, 40].

Hepcidin is a key regulator of systemic iron homeostasis, and its unbalanced production is responsible for the pathogenesis of various iron disorders [18]. A previous study showed that increased hepcidin production can be regulated by plasma and liver iron levels, which function as a feedback mechanism to maintain stable body iron levels [41]. Similar results were found in the present study: serum hepcidin levels were positively correlated with levels of serum ferritin and the change trend of these two indicators were similar, suggesting that the state of iron overload could promote serum hepcidin production. An experimental study conducted by Le Guenno et al. in mice revealed decreased hepcidin levels in a high-energy diet-induced insulin resistance model [42], which might be result of regulation of abnormal insulin by enhancing STAT3 protein synthesis and DNA-binding activity [13]. And also, a study conducted by Carvalho et al. found relatively higher levels of serum hepcidin in chronic periodontitis, which may be caused by chronic inflammatory stimulation [43]. Findings from these previous studies could further explain the lower hepcidin levels observed in the T2DM group compared with the CP group in the present study.

Since hepcidin is regulated by iron levels, the concentration of hepcidin alone could not effectively reflect the ability of the body to regulate iron load. The hepcidin/ferritin ratio has been reported to reflect the adequacy of hepcidin production for a given iron load; when this ratio decreases, the hepcidin produced is insufficient for the iron overload in the body [24, 44], which leads to subsequent iron accumulation in the tissue [45]. This might be the right time to introduce medications or other therapeutic interventions. In the present study, the decrease in the serum hepcidin/ferritin ratio in CP patients with T2DM suggested inadequacy of hepcidin in these patients. In addition, the hepcidin/ferritin ratio in the CP with T2DM group was significantly lower than that in the T2DM group, which indicated that CP might be a primary cause of the aggravation of hepcidin deficiency observed in T2DM patients.

CP is an inflammatory disease of the periodontal supporting tissues caused by microorganisms in the dental biofilm [46]. The majority of key periodontal pathogens possess haemolytic activity [11]. It is reported that between a third to three quarters of patients with periodontitis harbor β-haemolytic bacteria, which can lead to the dissolution of erythrocyte [47]. The iron overload might be caused by the invasion of periodontal pathogens. In the present study, we found CP was the risk factor of increased serum ferritin, increased serum hepcidin and decreased hepcidin/ ferritin ratio. Therefore, a hypothesis can be proposed that CP might be regarded as an independent risk factor for iron overload in the body and inadequate hepcidin production. Moreover, T2DM might be promoted by iron excess and insufficient hepcidin production [14]. So, a speculation is proposed that CP might promote T2DM development by inducing iron overload and inadequate production of hepcidin, which still need further explore. By correcting hepcidin levels, we can prevent cellular iron overload and reduce the risk of diabetes [48]. Some studies have shown that increased hepcidin levels might help reduce the incidence of T2DM. Thus, several hepcidin-modulating drugs are currently under development [49]. For example, Ramos E et al. found minihepcidins, small drug-like hepcidin agonists, could help reduce the body iron overload [45]. Such new drugs may, at least hypothetically, ameliorate the endocrinal diabetic functions by reducing tissue iron retention [50]. And also, previous studies showed that after nonsurgical periodontal therapy, CP patients showed decreased ferritin and prohepcidin (the prohormone of hepcidin) levels, suggesting that the iron overload and inflammatory burden had improved after treatment [35, 51]. The results from the present study might provide theoretical evidence regarding the importance of control of periodontitis by using periodontal therapy in patients who are at a high risk of T2DM or those with T2DM.

Whole saliva is composed of secretions from major and minor salivary glands as well as the gingival crevicular fluid [25]. Human saliva is a rich reservoir of biological markers that monitor systemic conditions. Mythily et al. reported that T2DM-associated serum proteins could be detected mostly in saliva, which might be useful for T2DM screening [52]. On the other hand, Abdolsamadi et al. indicated that salivary melatonin plays a vital role in the pathogenesis of diabetes and periodontal diseases and might become a key biomarker in the diagnosis and treatment of these two diseases [53]. However, to our knowledge, no study has explored salivary indicators that can reflect iron metabolism disorder in T2DM patients with or without CP. In the present study, salivary ferritin levels were significantly higher in CP and T2DM groups compared with the control group. Curve fitting showed that the level of serum ferritin increased with salivary ferritin. Based on these results, salivary ferritin might be regarded as a reference for body iron load. Cicek et al. found that hepcidin was localized in the striated ducts of the sublingual and parotid glands, and salivary hepcidin levels (mean = 714.10 ng/mL, control group) were correlated with blood hepcidin levels [27]. Our study results showed that hepcidin can indeed be detected in the saliva, but its levels remained low (median = 1.12 ng/mL, control group). The wide difference between levels observed in our and in the previous study might be attributed to differences in participant age or race, which warrants further research for confirmation.

## Conclusions

Our results imply that Iron overload and hepcidin inadequacy existed in CP with T2DM patients. Furthermore, salivary ferritin level might provide a reference for body iron load. Additional studies are needed to explore other potential influential factors of salivary hepcidin concentrations.

## Abbreviations

ALT: Alanine transaminase
ANCOVA: Analysis of covariance
ANOVA: Analysis of variance
AST: Aspartate transaminase
BI: Bleeding index
BMI: Body mass index
CAL: Clinical attachment loss
CHOL: Cholesterol
CMIA: Chemiluminescence microparticle immunoassay
CP: Chronic periodontitis
ELISA: Enzyme-linked immunosorbent assay
FBG: Fasting blood glucose
HDL: High-density lipoprotein
HepG2: Hepatocellular carcinoma
LDL: Low-density lipoprotein
OR: Odds ratio
PD: Probing depth
PI: Plaque index
RLUs: Relative light units
STAT3: Signal transducers and activators of transcription 3
T2DM: Type 2 diabetes mellitus
TG: Triglyceride

## References

[1] Armitage GC (2004). Periodontal diagnoses and classification of periodontal diseases. Periodontology 2000.

[2] Expert Committee on the D, Classification of Diabetes M (2003). Report of the expert committee on the diagnosis and classification of diabetes mellitus. Diabetes care.

[3] Lalla E, Papapanou PN (2011). Diabetes mellitus and periodontitis: a tale of two common interrelated diseases. Nat Rev Endocrinol.

[4] Santacroce L, Monea A, Marrelli M, Man A. Oral candidiasis and inflammatory response: a potential synergic contribution to the onset of Type-2 diabetes mellitus. Australasian Medical Journal. 2017;10(6)

[5] Demmer RT, Jacobs DR, Desvarieux M (2008). Periodontal disease and incident type 2 diabetes: results from the first National Health and nutrition examination survey and its epidemiologic follow-up study. Diabetes Care.

[6] Preshaw PM, Alba AL, Herrera D, Jepsen S, Konstantinidis A, Makrilakis K, Taylor R (2012). Periodontitis and diabetes: a two-way relationship. Diabetologia.

[7] Taylor JJ, Preshaw PM, Lalla E (2013). A review of the evidence for pathogenic mechanisms that may link periodontitis and diabetes. J Periodontol.

[8] Novotna M, Podzimek S, Broukal Z, Lencova E, Duskova J (2015). Periodontal diseases and dental caries in children with type 1 diabetes mellitus. Mediat Inflamm.

[9] Santacroce L, Carlaio RG, Bottalico L (2010). Does it make sense that diabetes is reciprocally associated with periodontal disease?. Endocr Metab Immune Disord Drug Targets.

[10] Li S, Schmalz G, Schmidt J, Krause F, Haak R, Ziebolz D. Antimicrobial peptides as a possible interlink between periodontal diseases and its risk factors: a systematic review. J Periodontal Res. 2018;53(2):145-55. 10.1111/jre.12482 28990193

[11] Falkler WA, Clayman EB, Shaefer DF (1983). Haemolysis of human erythrocytes by the fusobacterium nucleatum associated with periodontal disease. Arch Oral Biol.

[12] Wang L, Duan XL, Wang YZ, Chang YZ, Qian ZM (2007). Progress of the study on iron disorder diseases. Sheng Li Ke Xue Jin Zhan.

[13] Yeap BB, Divitini ML, Gunton JE, Olynyk JK, Beilby JP, McQuillan B, Hung J, Knuiman MW (2015). Higher ferritin levels, but not serum iron or transferrin saturation, are associated with type 2 diabetes mellitus in adult men and women free of genetic haemochromatosis. Clin Endocrinol.

[14] Silva M, Bonomo Lde F, Oliveira Rde P, Geraldo de Lima W, Silva ME, Pedrosa ML (2011). Effects of the interaction of diabetes and iron supplementation on hepatic and pancreatic tissues, oxidative stress markers, and liver peroxisome proliferator-activated receptor-alpha expression. J Clin Biochem Nutr.

[15] Kumar J, Teoh SL, Das S, Mahakknaukrauh P (2017). Oxidative stress in oral diseases: understanding its relation with other systemic diseases. Front Physiol.

[16] Wang H, Li H, Jiang X, Shi W, Shen Z, Li M (2014). Hepcidin is directly regulated by insulin and plays an important role in iron overload in streptozotocin-induced diabetic rats. Diabetes.

[17] Vela D, Sopi RB, Mladenov M. Low Hepcidin in diabetes mellitus: examining the molecular links and their clinical implications. Can J Diabetes. 2017; 10.1016/j.jcjd.2017.04.007 28662967

[18] Zhao N, Zhang AS, Enns CA (2013). Iron regulation by hepcidin. J Clin Invest.

[19] Nemeth E, Tuttle MS, Powelson J, Vaughn MB, Donovan A, Ward DM, Ganz T, Kaplan J (2004). Hepcidin regulates cellular iron efflux by binding to ferroportin and inducing its internalization. Science.

[20] Poli M, Asperti M, Ruzzenenti P, Regoni M, Arosio P (2014). Hepcidin antagonists for potential treatments of disorders with hepcidin excess. Front Pharmacol.

[21] Fischer R, Piga A, Harmatz P, Nielsen P (2005). Monitoring long-term efficacy of iron chelation treatment with biomagnetic liver susceptometry. Ann N Y Acad Sci.

[22] Stuart-Smith SE, Hughes DA, Bain BJ (2005). Are routine iron stains on bone marrow trephine biopsy specimens necessary?. J Clin Pathol.

[23] Bah A, Pasricha S-R, Jallow MW, Sise EA, Wegmuller R, Armitage AE, Drakesmith H, Moore SE, Prentice AM (2017). Serum Hepcidin concentrations decline during pregnancy and may identify Iron deficiency: analysis of a longitudinal pregnancy cohort in the Gambia. J Nutr.

[24] Sam AH, Busbridge M, Amin A, Webber L, White D, Franks S, Martin NM, Sleeth M, Ismail NA, Daud NM (2013). Hepcidin levels in diabetes mellitus and polycystic ovary syndrome. Diabet Med.

[25] Wang Q, Yu Q, Lin Q, Duan Y (2015). Emerging salivary biomarkers by mass spectrometry. Clin Chim Acta.

[26] Canatan D, Akdeniz SK (2012). Iron and ferritin levels in saliva of patients with thalassemia and iron deficiency anemia. Mediterranean journal of hematology and infectious diseases.

[27] Cicek D, Dagli AF, Aydin S, Baskaya Dogan F, Dertlioglu SB, Ucak H, Demir B (2014). Does hepcidin play a role in the pathogenesis of aphthae in Behcet's disease and recurrent aphthous stomatitis?. J Eur Acad Dermatol Venereol.

[28] Jou YJ, Lin CD, Lai CH, Chen CH, Kao JY, Chen SY, Tsai MH, Huang SH, Lin CW (2010). Proteomic identification of salivary transferrin as a biomarker for early detection of oral cancer. Anal Chim Acta.

[29] American Diabetes A (2008). Diagnosis and classification of diabetes mellitus. Diabetes Care.

[30] Armitage GC (1999). Development of a classification system for periodontal diseases and conditions. Annals of periodontology.

[31] Muhlemann HR, Son S (1971). Gingival sulcus bleeding--a leading symptom in initial gingivitis. Helvetica odontologica acta.

[32] Silness J, Loe H (1964). Periodontal disease in pregnancy. Ii. Correlation between oral hygiene and periodontal Condtion. Acta Odontol Scand.

[33] Navazesh M (1993). Methods for collecting saliva. Ann N Y Acad Sci.

[34] Dixon SJ, Stockwell BR (2014). The role of iron and reactive oxygen species in cell death. Nat Chem Biol.

[35] Chakraborty S, Tewari S, Sharma RK, Narula SC (2014). Effect of non-surgical periodontal therapy on serum ferritin levels: an interventional study. J Periodontol.

[36] Chen LP, Chiang CK, Chan CP, Hung KY, Huang CS (2006). Does periodontitis reflect inflammation and malnutrition status in hemodialysis patients?. Am J Kidney Dis.

[37] Latha S, Thirugnanamsambandan S, Arun RT, Masthan KM, Malathi L, Rajesh E (2015). Serum ferritin level and red blood cell parameters in healthy controls and chronic periodontitis patients. J Pharm Bioallied Sci.

[38] Mandalunis P, Gibaja F, Ubios AM (2002). Experimental renal failure and iron overload: a histomorphometric study in the alveolar bone of rats. Exp Toxicol Pathol.

[39] Kimachi K, Miyoshi H (2012). Infectious disease associated with diabetes mellitus--mechanisms, classification, diagnosis and therapy. Nihon Rinsho.

[40] Neves JV, Wilson JM, Rodrigues PN (2009). Transferrin and ferritin response to bacterial infection: the role of the liver and brain in fish. Dev Comp Immunol.

[41] Ganz T (2011). Hepcidin and iron regulation, 10 years later. Blood.

[42] Le Guenno G, Chanseaume E, Ruivard M, Morio B, Mazur A (2007). Study of iron metabolism disturbances in an animal model of insulin resistance. Diabetes Res Clin Pract.

[43] Carvalho RC, Leite SA, Rodrigues VP, Pereira AF, Ferreira TC, Nascimento FR, Nascimento JR, Gomes-Filho IS, Bastos MG, Pereira AL (2016). Chronic periodontitis and serum levels of hepcidin and hemoglobin. Oral Dis.

[44] van Dijk BA, Laarakkers CM, Klaver SM, Jacobs EM, van Tits LJ, Janssen MC, Swinkels DW (2008). Serum hepcidin levels are innately low in HFE-related haemochromatosis but differ between C282Y-homozygotes with elevated and normal ferritin levels. Br J Haematol.

[45] Ramos E, Ruchala P, Goodnough JB, Kautz L, Preza GC, Nemeth E, Ganz T (2012). Minihepcidins prevent iron overload in a hepcidin-deficient mouse model of severe hemochromatosis. Blood.

[46] Ballini A, Cantore S, Farronato D, Cirulli N, Inchingolo F, Papa F, Malcangi G, Inchingolo AD, Dipalma G, Sardaro N (2015). Periodontal disease and bone pathogenesis: the crosstalk between cytokines and Porphyromonas Gingivalis. J Biol Regul Homeost Agents.

[47] Hillman JD, Maiden MF, Pfaller SP, Martin L, Duncan MJ, Socransky SS (1993). Characterization of hemolytic bacteria in subgingival plaque. J Periodontal Res.

[48] Vela D, Leshoski J, Gjorgievska ES, Hadzi-Petrushev N, Jakupaj M, Sopi RB, Mladenov M (2017). The role of insulin therapy in correcting Hepcidin levels in patients with type 2 diabetes mellitus. Oman Med J.

[49] Theurl I, Schroll A, Sonnweber T, Nairz M, Theurl M, Willenbacher W, Eller K, Wolf D, Seifert M, Sun CC (2011). Pharmacologic inhibition of hepcidin expression reverses anemia of chronic inflammation in rats. Blood.

[50] Liu J, Qian L, Guo L, Feng Y (2018). Studying hepcidin and related pathways in osteoblasts using a mouse model with insulin receptor substrate 1loss of function. Mol Med Rep.

[51] Vilela EM, Bastos JA, Fernandes N, Ferreira AP, Chaoubah A, Bastos MG (2011). Treatment of chronic periodontitis decreases serum prohepcidin levels in patients with chronic kidney disease. Clinics (Sao Paulo).

[52] Srinivasan M, Blackburn C, Mohamed M, Sivagami AV, Blum J (2015). Literature-based discovery of salivary biomarkers for type 2 diabetes mellitus. Biomark Insights.

[53] Abdolsamadi H, Goodarzi MT, Ahmadi Motemayel F, Jazaeri M, Feradmal J, Zarabadi M, Hoseyni M, Torkzaban P (2014). Reduction of melatonin level in patients with type II diabetes and periodontal diseases. J Dent Res, Dent Clin, Dent prospects.

